# Development of a Predictive Model of Difficult Hemostasis following Endobronchial Biopsy in Lung Cancer Patients

**DOI:** 10.1155/2019/1656890

**Published:** 2019-02-26

**Authors:** Saibin Wang

**Affiliations:** Department of Respiratory Medicine, Jinhua Municipal Central Hospital, No. 365, East Renmin Road, Jinhua 321000, Zhejiang Province, China

## Abstract

Endobronchial biopsy (EBB)-induced bleeding is fairly common; however, it can be potentially life-threatening due to difficult hemostasis following EBB. The aim of this study was to develop a predictive model of difficult hemostasis post-EBB. A total of 620 consecutive patients with primary lung cancer who had undergone EBB between 2014 and 2018 in a large tertiary hospital were enrolled in this retrospective single-center cohort study. Patients were classified into the difficult hemostasis group and the nondifficult hemostasis group according to hemostatic measures used following EBB. The LASSO regression method was used to select predictors and multivariate logistic regression was applied to develop the predictive model. The area under the curve (AUC) of the model was calculated. Bootstrapping method was applied for internal validation. Calibration curve analysis and decision curve analysis (DCA) were also performed. A nomogram was constructed to display the model. The incidence of difficult hemostasis post-EBB was 11.9% (74/620). Eight variables were selected by the LASSO regression analysis and seven (histological type of cancer, lesion location, neutrophil percentage, activated partial thromboplastin time, low density lipoprotein cholesterol, apolipoprotein-E, and pulmonary infection) of them were finally included in the predictive model. The AUC of the model was 0.822 (95% CI, 0.777-0.868), and it was 0.808 (95% CI, 0.761-0.856) in the internal validation. The predictive model was well calibrated and DCA indicated its potential clinical usefulness, which suggests that the model has great potential to predict lung cancer patients with a more difficult post-EBB hemostasis.

## 1. Introduction

Bleeding is a very common complication during endobronchial biopsy (EBB), and biopsy-induced difficult hemostasis not only affects further bronchoscopic procedures but also can be life-threatening [1–3]. Currently, the common biopsy modalities of EBB used include forceps biopsies, cryobiopsies, bronchial brushing, and needle aspiration biopsies [4], among which forceps biopsy is the most widely used biopsy methods in clinical practice [5]. Although several risk factors for bleeding during bronchoscopy have been proposed [6, 7], difficult hemostasis is often unexpected following biopsy.

Malignant tissue is more likely to bleed compared to benign tissue during bronchoscopy [8]. Patients with lung cancer frequently undergo bronchoscopy and the incidence of EBB-induced hemorrhage exceeds 30% in lung cancer patients [2]. Generally, most EBB-induced bleeding is self-stopping or hemostasis may be induced just by local intrabronchial instillation of hemostatic drugs, such as 4°C physiological saline or diluted adrenalin (1:10000-1:100000) [9]. However, difficult hemostasis following EBB may also occur, requiring the administration of argon plasma coagulation (APC), electrocoagulation, or endobronchial balloon tamponade to control bleeding [10].

Since uncontrolled endobronchial bleeding is still the main cause of death during bronchoscopy [1, 3, 11], a preoperative prediction for the occurrence of difficult hemostasis would help adjust biopsy modality, reduce the number of biopsies, and prepare hemostasis measures in advance, thereby improving the safety of EBB. However, to our knowledge, no relative predictive model is available to date. In the current study, clinical characteristics, tumor features, and laboratory tests of patients with lung cancer who had undergone EBB were retrospectively investigated to develop a predictive model of difficult hemostasis following EBB.

## 2. Materials and Methods

### 2.1. Study Population and Ethics Statement

This study was based on a single-center retrospective cohort study. A total of 620 lung cancer patients who had consecutively undergone EBB between January 2014 and February 2018 were enrolled at a 2600-bed tertiary hospital in this study. The study was approved by the institutional ethics committee of the hospital (No. 2018001007). Because all patient information used in this study was anonymous, patient informed consent was waived.

### 2.2. Variables Collection

In this cohort study, difficult hemostasis was defined as the requirement of APC or electrocoagulation for hemostasis following EBB; the remaining patients with either no bleeding, bleeding stopped on its own, or bleeding stopped with intrabronchial instillation of hemostatic drugs (4°C physiological saline or diluted adrenalin) were placed in the nondifficult hemostasis group. The following variables were collected from this study: patient's gender, age, brachial artery systolic pressure and diastolic pressure, weight, smoking history (yes or no), coexisting diabetes, COPD (chronic obstructive pulmonary disease) or CHD (coronary heart disease), pulmonary infection (yes or no); tumor features: lesions location, cancer histological type and stage (based on the TNM staging, the stage I-II was classified as early and stage III-IV, as advanced); laboratory tests on admission: white blood cell count, neutrophil percentage, neutrophil counts, hemoglobin, platelets, prothrombin time, activated partial thromboplastin time (APTT), aspartate aminotransferase, alanine aminotransferase, total cholesterol level, low density lipoprotein cholesterol (LDL-C), high density lipoprotein cholesterol (HDL-C), triglycerides, apolipoprotein-E, apolipoprotein-B and C-reactive protein (CRP).

### 2.3. Biopsy Procedures

The procedure (fiberoptic bronchoscopy, BF-1T60) was performed through a laryngeal mask airway under general anesthesia with the patients in the supine position. At the same location of the lesion 3-5 biopsies were usually performed using rigid endoscopic biopsy forceps. Generally, for obvious bleeding following EBB, intrabronchial instillation of 4°C physiological saline and/or diluted adrenalin (1:10000) was the first choice for hemostasis with repeats several times as needed. Electrocoagulation or APC was required when the bleeding failed to reach hemostasis by the means of the aforementioned drugs. All biopsies were performed by two experienced bronchoscopists.

### 2.4. Statistical Analysis

In this study, multiple imputation method was employed to account for missing data, and the baseline characteristics of the participants were summarized. Categorical variables are expressed as the number (percentage) and continuous variables as median (interquartile). Between two groups comparison, unpaired t-test or Kruskal-Wallis rank sum test, Pearson chi-squared test or the Fisher's exact test was performed as appropriate. The least absolute shrinkage and selection operator (LASSO) regression method was used for predictor selection and regularization. Multivariable logistic regression analysis using backward stepwise procedure and the likelihood ratio test on the basis of Akaike's information criterion (AIC) [12] were used to develop the predictive model. A nomogram was constructed to predict difficult hemostasis following EBB in lung cancer patients. The area under the curve (AUC) was calculated to determine the discriminatory capacity of the model, and internal validation was performed using bootstrapping (resampling = 1000) [13]. Calibration was tested using calibration plots and the Hosmer-Lemeshow test. Decision curve analysis (DCA) was conducted to assess the potential clinical usefulness of the model [14]. Statistical analysis was done using R software (version 3.5.1), and P value < 0.05 was considered statistically significant.

## 3. Results

Among the 620 patients, 74 (11.9%, 95% confidence interval [CI], 9.4%-14.5%) experienced post-EBB difficult hemostasis, and no patient died of severe bleeding after electrocoagulation and APC hemostasis. Smoking, pulmonary infection, histological types of cancer, lesion location, neutrophil percentage, CRP, APTT, HDL-C, alanine aminotransferase, and apolipoprotein-E were statistically different as assessed by univariate analysis. Patient's baseline characteristics, tumor features, and laboratory tests are shown in Table 1.

**Figure 1:**
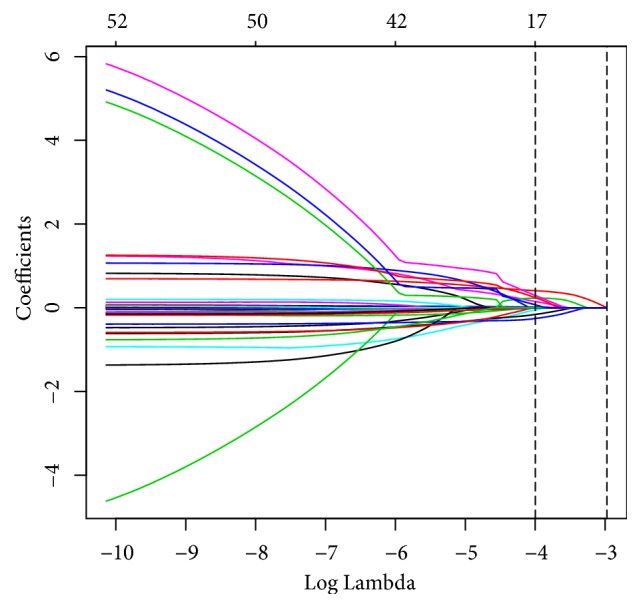
Variables selection using the LASSO regression analysis with 10-fold cross-validation. Coefficients were produced against the log lambda sequence. Dotted vertical line was drawn according to the minimum criteria (left dotted line), and a total of 8 nonzero coefficients were filtered and used to construct predictive model.

Of the 31 variables, 8 variables were filtered based on nonzero coefficients calculated by the LASSO regression analysis using the minimum criteria (Figure 1). These variables were lesions location, cancer histological type, pulmonary infection, neutrophil percentage, APTT, HDL-C, LDL-C, and apolipoprotein-E.

The aforementioned 8 predictors were included in multivariable logistic regression analysis. Backward stepwise selection was applied to develop a predictive model and 7 predictors (excluding HDL-C) were eventually incorporated into the model according to the likelihood ratio test with AIC.

**Figure 2:**
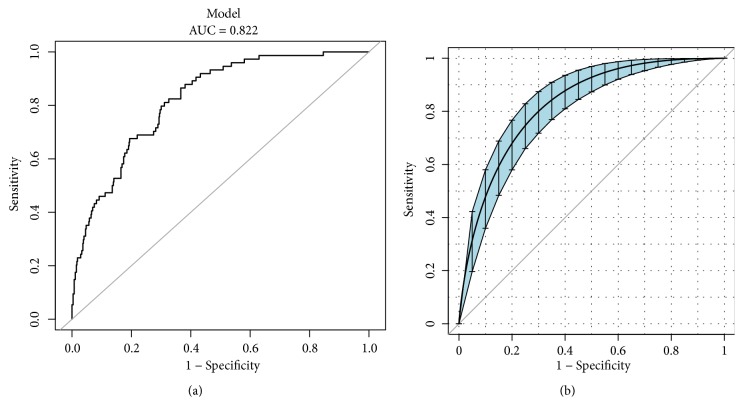
The AUC represents the discriminatory ability of the model. (a) shows AUC of the predictive model and (b) shows AUC of the internal validation with the bootstrap method (resampling times = 1000). The dotted vertical lines represent 95% confidence interval. AUC, area under the curve.

**Figure 3:**
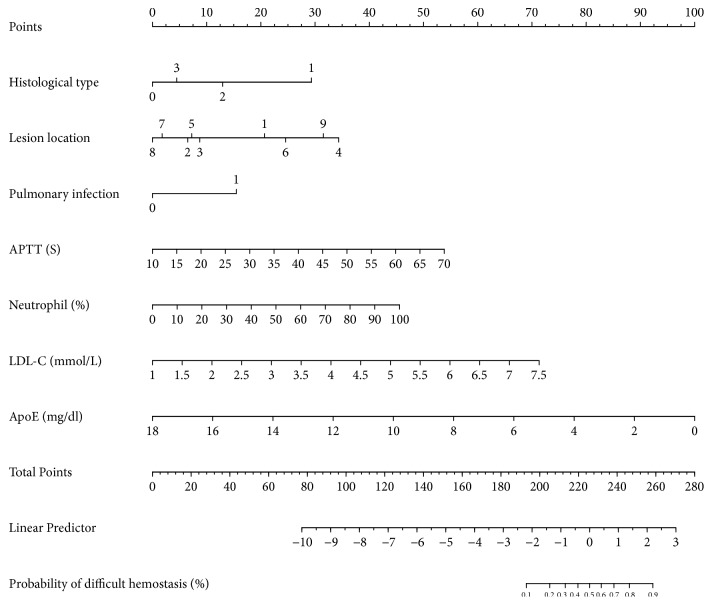
Nomogram for difficult hemostasis following endobronchial biopsy in lung cancer patients. Firstly, find point for each predictor of an individual on the uppermost rule. Secondly, add all points together and find the “total points” on rule. At last, the corresponding predicted probability of difficult hemostasis following endobronchial biopsy could be found on the lowest rule. Codes annotation: histological type of lung cancer: 0, adenocarcinoma; 1, squamous cell carcinoma; 2, small-cell lung carcinoma; 3, other types. Lesion location: 1, left main bronchi; 2, left upper lobar bronchi; 3, left lower lobar bronchi; 4, right main bronchus; 5, right upper lobar bronchi; 6, right middle bronchus; 7, right middle lobar bronchi; 8, right lower lobar bronchi; 9, the trachea. Pulmonary infection: 0, no; 1, yes. APTT, activated partial thromboplastin time; LDL-C, low density lipoprotein cholesterol; ApoE, apolipoprotein-E.

The AUC for the predictive model reached was 0.822 (95% CI, 0.777-0.868), and it was 0.808 (95% CI, 0.761-0.856) in the internal validation using bootstrapping (resampling times = 1000) (Figure 2). A nomogram was constructed to display the predictive model (Figure 3), providing a quantitative tool to predict the probability of difficult hemostasis following EBB.

**Figure 4:**
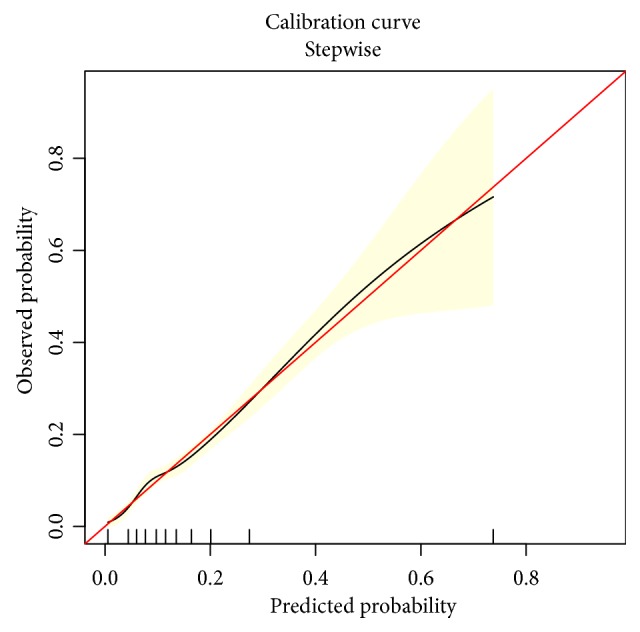
Calibration curve of the predictive model. It shows a good fit between the predicted risks of difficult hemostasis following endobronchial biopsy and observed outcomes in patients with lung cancer. The red solid line represents an ideal predictive model, and the solid black line shows the actual performance of the predictive model. The yellow shadow represents 95% confidence interval. The Hosmer-Lemeshow test yielded a P value of 0.985, an Emax of 0.027, and an Eavg of 0.006.

As is shown in Figure 4, the model was well calibrated. A nonsignificant statistical value (P = 0.985) was yielded in the Hosmer-Lemeshow test with an Emax value of 0.027 and Eavg value of 0.006, indicating that there was no departure from a perfect fit between predicted and observed values.

**Figure 5:**
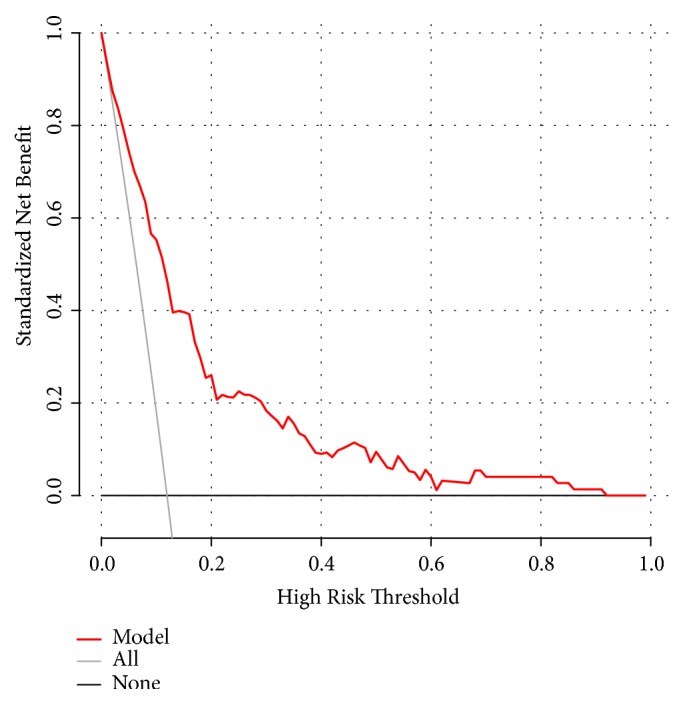
Decision curve of the predictive model. Net benefit was produced against the high risk threshold. The red solid line represents the predictive model. The decision curve indicates that when the threshold probability is less than 90%, application of this predictive model would add net benefit compared with either the treat-all or the treat-none strategies.

The decision curve (Figure 5) demonstrated that application of this model to predict post-EBB difficult hemostasis would add more benefit compared with either the treat-all or the treat-none strategies. Specifically, when the threshold probability of difficult hemostasis following EBB is < 90% based on the predictive model, the clinical use of this predictive model can benefit lung cancer patients undergoing EBB.

## 4. Discussion

In the present study, a predictive model of difficult hemostasis following EBB was developed. This model incorporated 7 predictors, including histological type of cancer, lesion location, pulmonary infection, neutrophil percentage, APTT, LDL-C, and apolipoprotein-E. The model showed good discriminatory ability in the derivation cohort and in the internal validation. The model was well-calibrated and showed potential clinical usefulness.

EBB-induced bleeding has always been a common concern for bronchoscopists [1, 3, 11]. Unexpected difficult hemostasis could occur during EBB leading to severe bleeding that may prove being life-threatening [3, 15]. It has been reported that the incidence of bleeding during bronchoscopy ranges from <1% to 20% [16]. However, bleeding risk significantly increases when transbronchial biopsies are performed [17]. Additionally, the incidence of biopsy-induced bleeding is related to the biopsy tissue. When compared with benign mucosal lesions, malignant tissue is more susceptible to bleeding following EBB [2, 8]. It should also be noted that lung cancer patients have become the main population receiving EBB.

In previous studies, several risk factors for bleeding during bronchoscopy had been proposed, such as immunosuppression, thrombocytopenia, anticoagulant drug use, or coagulation dysfunction [6, 7]. However, it is still difficult to predict the occurrence of difficult hemostasis after a biopsy in patients who do not have a significant risk of bleeding. To our knowledge, no predictive model for post-EBB difficult hemostasis is available to date.

For bronchoscopists, to predict intraoperative EBB-induced bleeding risk can help guide their preoperative clinical decision making and select appropriate hemostasis measures during EBB. Currently, commonly used hemostasis measures during EBB include intrabronchial instillation of 4°C physiological saline and/or diluted adrenalin, which is suitable for hemostasis with microbleeding or mild-bleeding [18]. For moderate-bleeding or difficult-to-stop bleeding after repeated use of the aforementioned drugs, electrocoagulation, APC and intravenous infusion of pituitrin are usually required. In case of massive bleeding, endobronchial balloon tamponade for persistent hemoptysis and surgery may be needed [10, 19]. Because of its frequent occurrence and potential hazard, difficulty in hemostasis following EBB was the main focus of this study.

The predictive model for post-EBB difficult hemostasis in the present study was developed based on 7 predictors, including histological type of cancer, lesion location, pulmonary infection, neutrophil percentage, APTT, LDL-C, and apolipoprotein-E. These predictors were filtered by LASSO regression analysis, which was considered to surpass the technique of choosing predictors based on their univariable association strength with outcome [20, 21]. Additionally, all 7 predictors are readily accessible clinically. The prediction model showed both good discrimination ability and calibration. DCA is a recommended novel method for evaluating the clinical value of a predictive model [22, 23]. The decision curve based on this model revealed that when the threshold probability of a subject was < 90%, applying this model to predict post-EBB difficult hemostasis would benefit when compared to either treat-all or treat-none strategies. In addition, a nomogram was also constructed to facilitate the application of the model.

Some limitations of this predictive model are worth noting. Firstly, this model was constructed based on a single-center retrospective study, which inevitably suffered from confounding bias; for example, indication of the specific tools (APC, electrocoagulation, or endobronchial balloon tamponade) used for the stop bleeding is related to the decision of the physician, which is difficult to be reconciled among bronchoscopists. Secondly, an independent validation is very important for determining the clinical usefulness of a predictive model; therefore, whether the proposed model is applicable to other endoscopic centers needs further validation. Thirdly, the mechanism underlying some predictors in bleeding is still unclear, such as LDL-C. In addition, during hemostasis, the time and frequency of use of electrocoagulation and/or APC may further distinguish the degree of difficulty in hemostasis; however, these variables were not available in the original data. Despite these limitations, the present study is the first to develop a predictive model for difficult hemostasis following EBB.

## 5. Conclusion

A difficult hemostasis risk prediction model for EBB-induced bleeding in lung cancer patients was developed, which incorporated 7 readily available clinical variables. This model showed good discriminatory ability and potential clinical usefulness, and thus it may be of great value to facilitate the prediction and management of EBB-induced difficult hemostasis.

## Figures and Tables

**Table 1:** Baseline characteristics and blood tests of the study participants.

Variables	Difficult hemostasis		P value
	No (n = 546)	Yes (n = 74)	
Gender, n (%)			0.038
Female	124 (22.71)	9 (12.16)	
Man	422 (77.29)	65 (87.84)	
Age (years)	65 (59-70)	65 (59-70)	0.757
Smoking, n (%)			0.003
No	214 (39.19)	16 (21.62)	
Yes	332 (60.81)	58 (78.38)	
SBP (mmHg)	131 (119-145)	128 (111-143)	0.184
DBP (mmHg)	78 (70-86)	70 (70-88)	0.326
Weight (kg)	60 (53-66)	60 (53-70)	0.353
COPD, n (%)			0.738
No	511 (93.59)	70 (94.59)	
Yes	35 (6.41)	4 (5.41)	
Diabetes, n (%)			0.410
No	516 (94.51)	72 (97.30)	
Yes	30 (5.49)	2 (2.70)	
CHD, n (%)			0.722
No	529 (96.89)	71 (95.95)	
Yes	17 (3.11)	3 (4.05)	
Pulmonary infection, n (%)			<0.001
No	340 (62.27)	28 (37.84)	
Yes	206 (37.73)	46 (62.16)	
Cancer stage, n (%)			0.824
Early	295 (54.03)	41 (55.41)	
Advanced	251 (45.97)	33 (44.59)	
Histological types, n (%)			<0.001
Adenocarcinoma	161 (29.49)	5 (6.76)	
Squamous cell carcinoma	254 (46.52)	59 (79.73)	
SCLC	101 (18.50)	8 (10.81)	
Others	30 (5.49)	2 (2.70)	
Lesion location, n (%)			<0.001
Left main bronchus	29 (5.31)	8 (10.81)	
Left upper lobar bronchi	129 (23.63)	13 (17.57)	
Left lower lobar bronchi	98 (17.95)	10 (13.51)	
Right main bronchus	16 (2.93)	8 (10.81)	
Right upper lobar bronchi	137 (25.09)	14 (18.92)	
Right middle bronchus	21 (3.85)	8 (10.81)	
Right middle lobar bronchi	27 (4.95)	2 (2.70)	
Right lower lobar bronchi	84 (15.38)	7 (9.46)	
The trachea	5 (0.92)	4 (5.41)	
Hospitalization, (days)	11 (7-15)	10 (7-14)	0.360
WBC (×10^9^/L)	6.80 (5.45-8.50)	6.89 (5.43-8.88)	0.914
Neutrophil (%)	69.2 (61.8-75.6)	72.6 (65.8-80.9)	0.004
Neutrophils (×10^9^/L)	4.54 (3.50-6.35)	5.10 (3.82-6.70)	0.787
Hemoglobin (g/dl)	128 (116-140)	126 (112-137)	0.256
Platelets (×10^9^/L)	222 (173-280)	245 (171-317)	0.086
CRP (mg/L)	5.9 (1.1-31.8)	18.66 (3.8-44.5)	<0.001
PT (s)	13.0 (12.1-13.6)	13.3 (12.2-13.9)	0.663
APTT (s)	34.8 (31.5-38.2)	36.2 (33.0-41.2)	0.034
ALT (IU/L)	17.0 (12.0-26.0)	15.1 (12.0-21.4)	0.184
AST (IU/L)	23.0 (19.0-29.0)	23.0 (18.0-27.1)	0.233
Homocysteine (*μ*mol/L)	13.3 (10.7-16.6)	12.7 (10.1-15.7)	0.627
Triglyceride (mmol/L)	1.08 (0.79-1.46)	0.96 (0.71-1.15)	0.006
TC (mmol/L)	4.10 (3.46-4.77)	4.06 (3.53-4.77)	0.720
HDL-C (mmol/L)	1.14 (0.94-1.37)	1.07 (0.92-1.22)	0.072
LDL-C (mmol/L)	2.75 (2.26-3.30)	2.86 (2.42-3.40)	0.074
Apolipoprotein-B (g/L)	0.96 (0.77-1.18)	0.95 (0.80-1.15)	0.425
Apolipoprotein-E (mg/dL)	3.60 (2.90-4.80)	2.95 (2.40-3.98)	0.006

SBP, systolic blood pressure; DBP, diastolic blood pressure; COPD, chronic obstructive pulmonary disease; CHD, coronary heart disease; SCLC, small-cell lung carcinoma; WBC, white blood cell; CRP, C-reactive protein; PT, prothrombin time; APTT, activated partial thromboplastin time; ALT, alanine aminotransferase; AST, aspartate aminotransferase; TC, total cholesterol; HDL-C, high density lipoprotein cholesterol; LDL-C, low density lipoprotein cholesterol.

## Data Availability

The data used to support the findings of this study are available from the corresponding author upon request.
